# Single injection of very mild dose botulinum toxin in the vastus lateralis improves testicular spermatogenesis and sperm motility in ageing experimental mice

**DOI:** 10.1186/s42826-022-00117-4

**Published:** 2022-03-04

**Authors:** Risna Kanjirassery Radhakrishnan, Sowbarnika Ravichandran, Aishwarya Sukesh, Balamuthu Kadalmani, Mahesh Kandasamy

**Affiliations:** 1 Laboratory of Stem Cells and Neuroregeneration, Department of Animal Science, School of Life Sciences, Bharathidasan University, Tiruchirappalli, Tamil Nadu 620024 India; 2 Department of Animal Science, School of Life Sciences, Bharathidasan University, Tiruchirappalli, Tamil Nadu 620024 India; 3 Faculty Recharge Programme, University Grants Commission (UGC-FRP), New Delhi, 110002 India

**Keywords:** BoNT, Acetylcholine, Spermatogenesis, Testis, Sperm, Antioxidants

## Abstract

**Background:**

Botulinum toxin (BoNT) is a widely used therapeutic agent that blocks the excessive release of acetylcholine at the neuromuscular junction. Previously, repeated intracremasteric injections and slight overdose of BoNT have been reported to induce adverse effects in the testicular parameter of experimental rodents. However, a mild dose of BoNT is highly beneficial against skin ageing, neuromuscular deficits, overactive urinary bladder problems, testicular pain and erectile dysfunctions. Considering the facts, the possible therapeutic benefits of BoNT on the testis might be achieved at a very minimal dosage and via a distal route of action. Therefore, we revisited the effect of BoNT, but with a trace amount injected into the vastus lateralis of the thigh muscle, and analyzed histological parameters of the testis, levels of key antioxidants and sperm parameters in ageing experimental mice.

**Results:**

Experimental animals injected with 1 U/kg bodyweight of BoNT showed enhanced spermatogenesis in association with increased activities of key antioxidants in the testis, leading to enhanced amount of the total sperm count and progressive motility.

**Conclusions:**

This study signifies that a mild intramuscular dose of BoNT can be considered as a potent treatment strategy to manage and prevent male infertility.

## Background

Acetylcholine (ACh) is a key neuromodulator of the cholinergic system and biochemical regulator of muscle contraction [1]. ACh plays an important role in steroidogenesis and spermatogenesis in the testis [2, 3]. Abnormal ageing, psychological complications, neuropathogenic and altered metabolic conditions that are associated with the elevated levels of ACh and enhanced cholinergic signaling have been reported to induce endocrine imbalance, erectile failure and testicular defects leading to male infertility [4–7]. Therefore, blockade of excessive ACh release can be considered to restore male reproductive physiology that is lost during ageing and various diseases. Botulinum toxins (BoNTs) are a class of fatal proteins, mainly produced by *Clostridium botulinum*, which causes muscle paralysis as they act by binding presynaptically to high-affinity recognition sites on the cholinergic nerve terminals and effectively prevents the exocytosis of ACh vesicles at the neuromuscular junctions [8–10]. While a trace amount of purified BoNT has been identified to yield long-lasting anti-ageing therapeutic benefits and mitigating neuromuscular defects in various illnesses [8, 9, 11], some toxicological reports indicated that cremasteric injection of BoNT (10–40 U/kg) induces cell death and impairs spermiogenesis in the testis of experimental animals [12, 13]. However, a recent report revealed that intrascrotal injection of BoNT in adult men did not induce any obvious adverse effect [14]. Nevertheless, ample scientific evidence unequivocally points towards the positive episodes of a mild dose of BoNT against various clinical complications including movement disorders, cognitive deficits and behavioural abnormalities [8, 9, 11, 15–17]. Eventually, therapeutic forms of BoNTs have been used as a potent treatment agent against skin ageing, hyperhidrosis, chronic migraine, strabismus, movement disorders, overactive urinary bladder problems, testicular pain and erectile dysfunctions [8, 9, 18–20]. Notably, mild doses of therapeutic BoNTs have been identified to mitigate oxidative stress and facilitate cytoprotection in various tissues including the brain [11, 21–23]. Thus, we speculate that the reported adverse effect of BoNT on the testicular parameters in experimental animals might be largely due to slightly high dose at the proximal site and also by repeated injections. Considering the facts, the possible therapeutic benefits of BoNT on the testis might be achieved at its minimal dose and via distal route of action [11, 24]. Hence, we revisited the effect of BoNT on the histological parameters of the testis, status of key antioxidants, and sperm count and motility in ageing experimental animals. In this study, ageing experimental male mice were injected with a single dose of 1 U BoNT per Kg bodyweight into the vastus lateralis of the thigh. After four weeks of time interval, experimental mice were sacrificed and histological parameters of spermatogenesis, sperm count and motility were measured in corroboration with biochemical assessments of antioxidant levels in testis.

## Results

### BoNT treatment protects against the ageing-mediated decline in the total sperm count, motility and their morphological defects in experimental mice

**Fig. 1:**
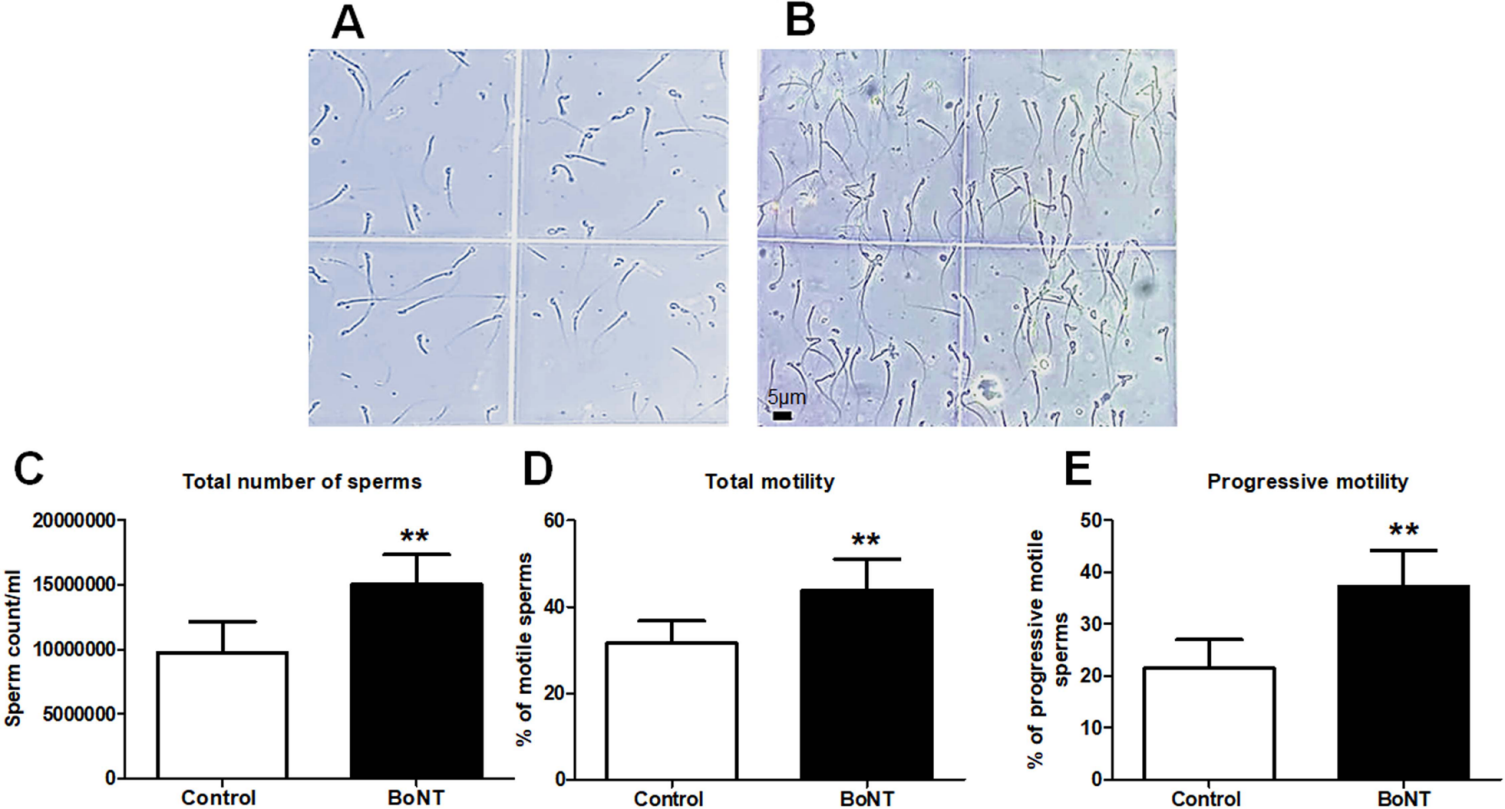
BoNT treatment improves sperm count and motility. Phase-contrast microscopic images of sperms obtained from the cauda epididymis of the control (**A**) and BoNT (**B**) treated animals. The bar graphs represent the total sperm count (**C**) and the percentage of total motility (**D**) and progressive motility (**E**) from the control and BoNT treated animals

**Fig. 2:**
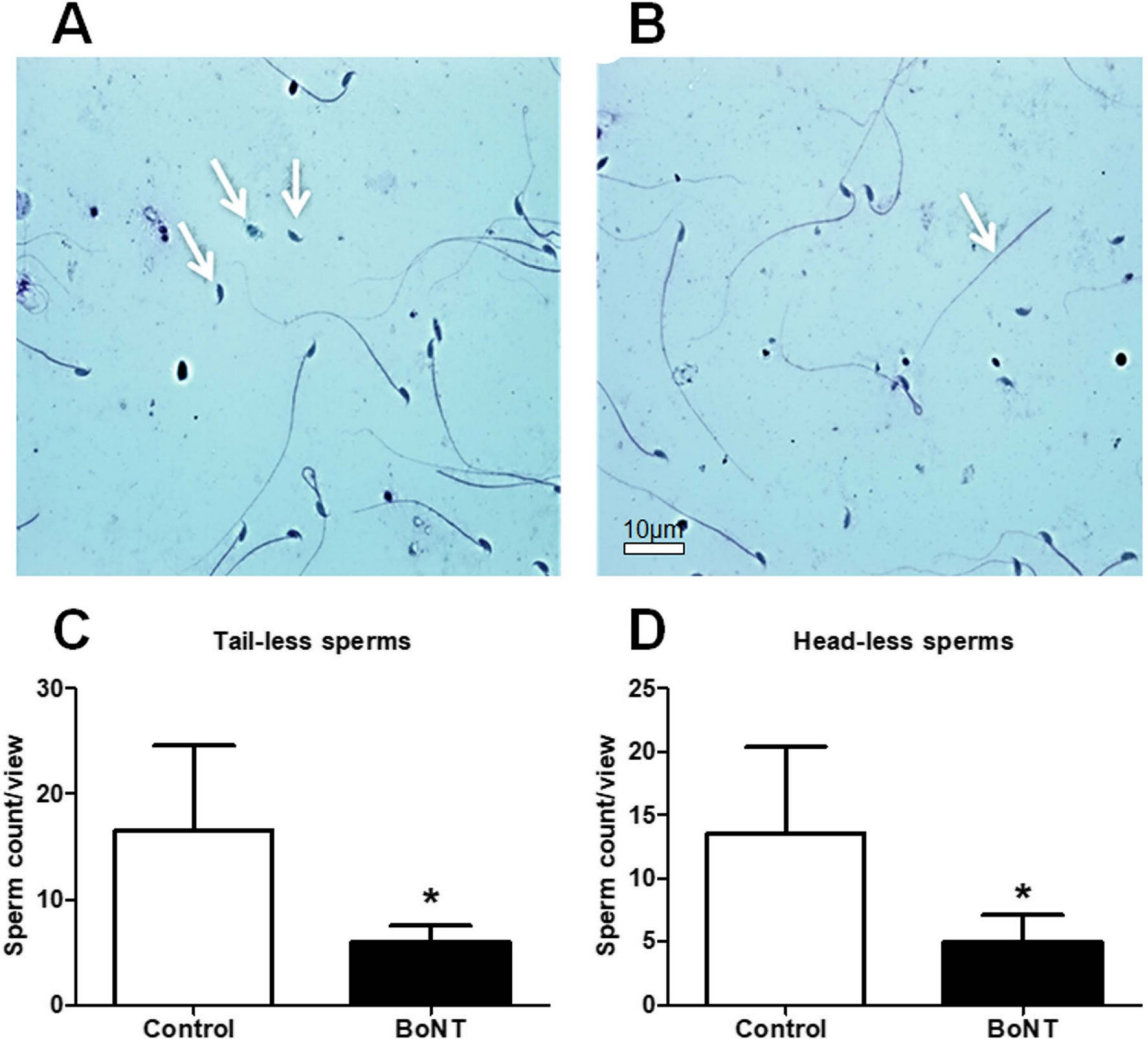
BoNT treatment reduces the morphological defects of the sperm. The light microscopic representative images of tailless (**A**) and headless (**B**) sperms from control animals. The bar graphs represent the number of tailless sperms (**C**) and headless (**D**) sperms in the control and BoNT treated animals

The estimation of the number of sperms revealed a significant increase in the total sperm count in the BoNT treated group compared to the control group (Control: 9,725,000 ± 2,421,260 vs BoNT: 15,037,500 ± 2,309,897). The total percentage of the motile sperms (Control: 31.5 ± 5.2 vs BoNT: 43.8 ± 7.8) and the percentage of sperms with progressive motility (Control: 21.5 ± 5.5 vs BoNT: 37.3 ± 6.8) were found to be increased in the BoNT treated group when compared to that of the control group (Fig. 1). In the morphological analysis, the number of tailless sperms (Control: 16.5 ± 4.8 vs BoNT: 6 ± 1.5) as well as headless sperms (Control: 13.5 ± 6.8 vs BoNT: 5 ± 2.8) were found to be minimized in the BoNT treated group than that of the control group (Fig. 2). However, no other obvious differences related to the morphology of sperms were noticed between the control and the BoNT treated group.

### BoNT treatment promotes the spermatogenic capacity in ageing experimental mice

**Fig. 3:**
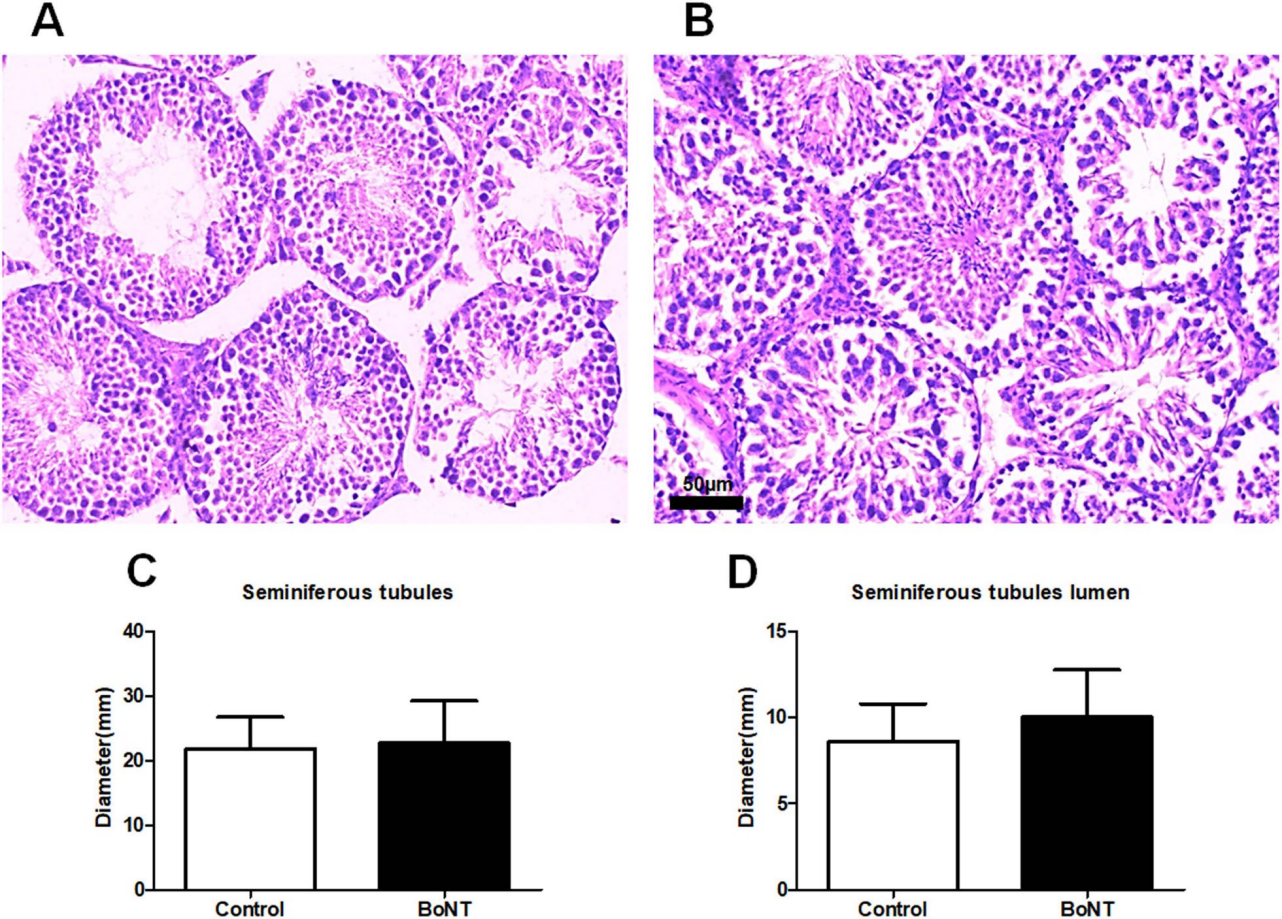
No major structural difference in the seminiferous tubules between control and BoNT treated animals. The H and E staining represents the morphology of seminiferous tubules in the testis of control (**A**) and BoNT treated animals (**B**). The bar graph data represents the diameter of seminiferous tubules (**C**), the diameter of lumen of seminiferous tubules (**D**)

**Fig. 4:**
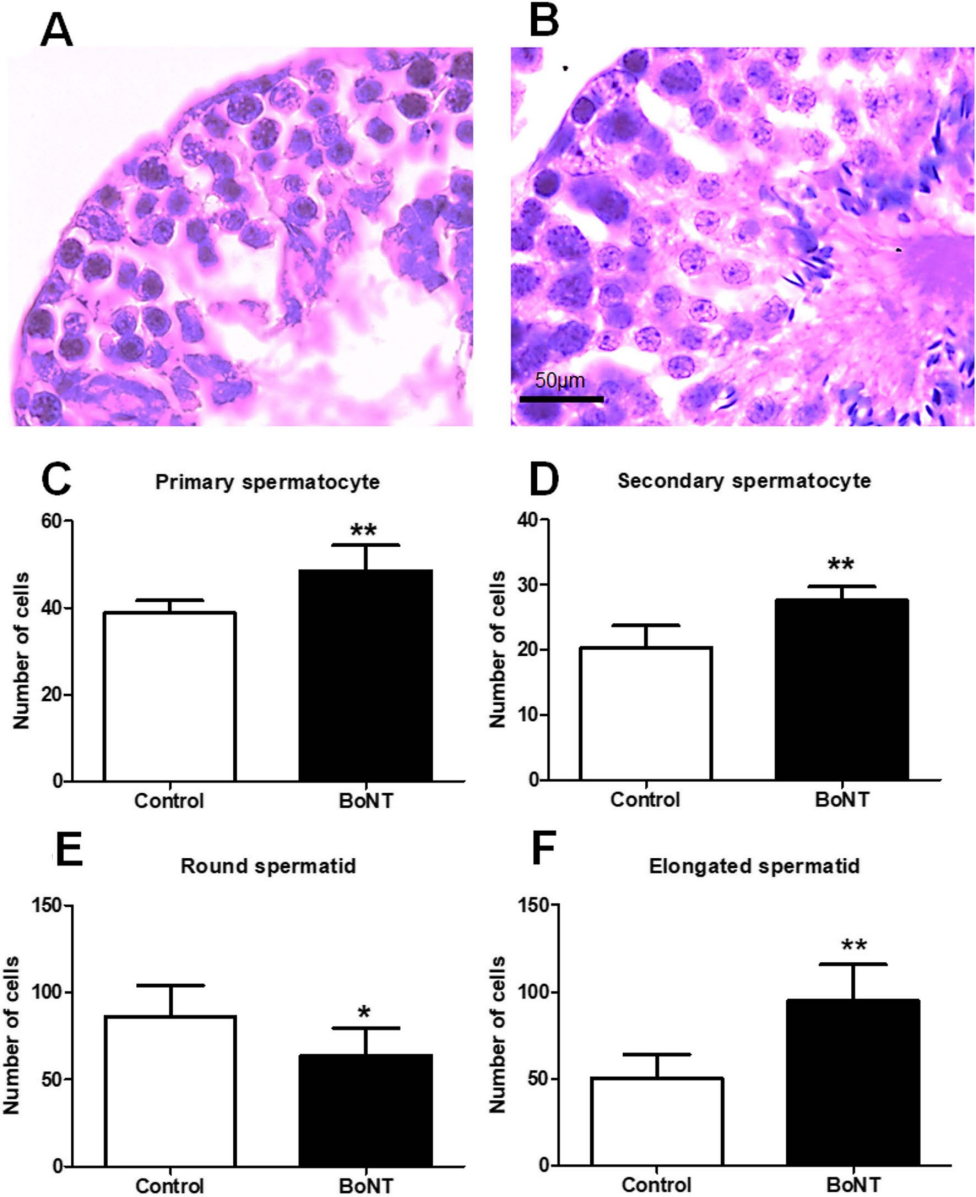
BoNT treatment indicates increase of spermatogenesis. Light microscopic images of the H and E staining depicts the cross sections of the seminiferous tubules in the control (**A**) and BoNT treated animals (**B**). The bar graphs show the total number of primary spermatocytes (**C**), secondary spermatocytes (**D**), round spermatids (**E**), and elongated spermatids (**F**) per cross section of the seminiferous tubule in the testis of control and BoNT treated animals

The histological examination of testicular cross-sections revealed no significant differences in the diameter of the seminiferous tubules between the control group and BoNT treated group (Control: 21.8 ± 6 vs BoNT: 22.6 ± 2). Also, the diameter of the lumen of the seminiferous tubule was almost similar among both the control and BoNT injected animals (Control: 8.5 ± 4.8 vs BoNT: 6.8 ± 1.9) (Fig. 3). Strikingly, the histological estimates of different cellular entities in the seminiferous tubules revealed a significant increase in the number of primary spermatocytes (Control: 39 ± 3 vs BoNT: 49 ± 6), secondary spermatocytes (Control: 20 ± 3 vs BoNT: 28 ± 2) and elongated spermatids (Control: 50 ± 14 vs BoNT: 99 ± 22) in the testis of the BoNT treated group than that of the control group. However, the estimated number of round spermatids (Control: 86 ± 17 vs BoNT: 63 ± 16) was found to be reduced in the testis of the BoNT treated group than the control group (Fig. 4).

### BoNT treatment increases the enzymatic activities of testicular antioxidants in ageing experimental mice

**Fig. 5:**
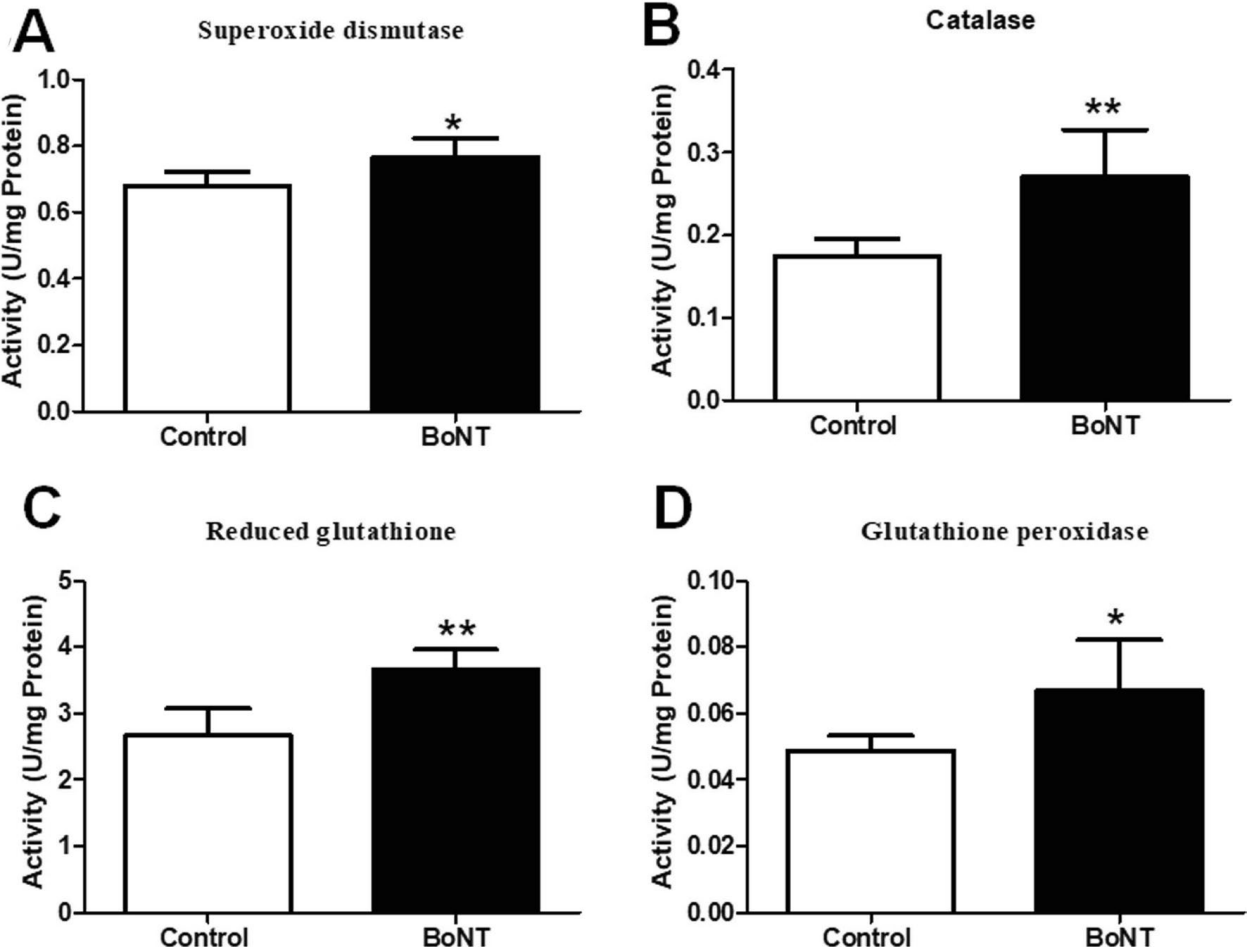
BoNT treatment increases the activities of key antioxidants in the testis. The bar graph represents the antioxidant activity of SOD (**A**), catalase (**B**), reduced glutathione (**C**) and glutathione peroxidase (**D**) in U/mg protein from the testicular extracts of control and BoNT treated animals

In the biochemical assessment of testicular protein extracts, the antioxidant activities of superoxide dismutase (SOD) (Control: 0.67 ± 0.04 vs BoNT: 0.76 ± 0.05), catalase (Control: 0.17 ± 0.02 vs BoNT: 0.27 ± 0.05), reduced glutathione (GSH) (Control: 2.6. ± 0.40 vs BoNT: 3.6 ± 0.29), and glutathione peroxidase (GPx) (Control: 0.40 ± 0.004 vs BoNT: 0.60 ± 0.01) were found to be significantly increased in the BoNT treated group compared to the control group (Fig. 5).

## Discussion

The present study demonstrates that a trace amount of BoNT injection at the distal intramuscular site in ageing experimental animals significantly improved the morphological and cytological characteristics of the seminiferous tubule, and the quantity and motility of sperms in association with the enhanced activities of testicular antioxidant enzymes. Recently, BoNT has been reported to facilitate increased locomotion, cognitive functions and reduced anxiety-related behaviours in ageing experimental rodents and in humans [11, 23, 25, 26]. It has been reported that BoNT treatment improves the blood circulation and oxygen supply, thereby providing trophic support to cell cycle events and cryoprotection in various organs [27, 28]. Considering the fact, the intramuscular injection of BoNT can be predicted to enhance the blood circulation in the testis of the experimental animals, which can promote the histological parameters and physiological functions of the testis. While the BoNT treated animals showed a significant increase in the number of primary and secondary spermatocytes, and elongated spermatids, a reduction in the number of round spermatids was evident in BoNT treated animals. The latter could be due to the enhanced differentiation of round to elongated spermatids contributing to an enhanced spermiogenesis in BoNT treated group, as BoNT has been reported to facilitate cellular differentiation in different tissues [29, 30].

Spermiogenesis is the cellular process of the testis by which haploid round spermatids undergo a series of events to become motile spermatozoa [31, 32]. Spermiogenesis commences after spermatocytes have accomplished the meiotic reductive cycle [33]. This process involves multifaceted morphological, biochemical, and physiological alterations in spermatids [31, 33, 34]. The major events in this process involve embellishment of the acrosome from the Golgi apparatus, condensation of the chromatin, centrosome disintegration, elimination of redundant cytoplasmic portion and formation of flagellum that are responsible for the motility of the spermatozoa [35, 36]. Thus, the molecular changes associated with BoNT treatment may involve the progression of the flagellum, differentiation of spermatozoa in the testis and increased motility of sperms in the epididymis. Therefore, future studies are required to exactly reveal the possible mechanisms by which BoNT injection facilitates spermatogenesis and sperm motility.

According to the free radical theory of ageing, reactive oxygen species (ROS)-mediated oxidative stress and mitochondrial damage in various tissues and organs represents an underlying biological cause of progressive ageing [37, 38]. Though generation and maintenance of the optimal levels of ROS can be important for the development and physiological functions of organs, unprecedented increased accumulation of ROS and failure in antioxidant defence would result in dysfunction of organs leading to diseases [37–39]. Among various organs, the testis appears to be highly vulnerable to abrupted levels of ROS [40, 41]. Considerable clinical and experimental reports established a clear notion that the high level of ROS could impair steroidogenesis and spermatogenesis in the testis [40]. Oxidative stress has typically been associated with the loss of structural and functional integrity of spermatozoa by inducing damages to DNA, RNA transcripts and telomeres, thereby leading to testicular atrophy and infertility in ageing and disease [38, 42, 43]. Oxidative stress in the testis contributes to low sperm count, impaired motility and abnormal morphology [44, 45]. Several studies have reported that ageing results in excessive production of ROS, reduction in antioxidants and DNA damage in spermatozoa, contributing to poor sperm count and motility [46–49]. Notably, increased levels of ROS has been reported to suppress the circulating concentration of the male sex hormones leading to abnormal hypothalamic–pituitary–gonadal (HPG) axis and infertility related issues [50]. Thus, neutralization of abruptly increased levels of ROS by the implementation of pharmacological agents has been considered as a potent therapeutic regime for infertility in order to restore the testicular functions [51, 52]. Earlier studies have indicated that the supplementation of antioxidants such as vitamin C, vitamin E and glutathione improves spermatogenesis and sperm quality [53, 54]. Recent studies have demonstrated that the use of therapeutic BoNT reduced oxidative stress in animal models and plasma of patients [22, 55, 56].

While the physiological levels of ACh is important for spermatogenesis and sperm motility [57], the abnormal concentration of ACh appears to induce oxidative stress in the testis, leading to and reduction in the motility and functioning of sperms [58, 59]. Notably, administration of ACh in the inferior spermatic nerve plexus, a site that regulates steroidogenesis, has been reported to block the secretion of testosterone that might be associated with reduced spermatogenesis in the testis [7]. Besides, Favaretto et al. reported that abnormal levels of endogenous ACh can interfere with the production of testosterone secretion in rat Leydig cells [60]. Similarly, cholinergic agonists have been reported to mediate inhibition of testosterone biosynthesis in the testis [61]. Therefore, it can be speculated that BoNT treatment might contribute directly to diminish the level of ROS or it may facilitate the production of antioxidants to counteract the free radicals in the testis. Indeed, the anti-ageing properties of BoNT injection in aesthetic rejuvenation and course of action in cosmetics have been considered to be mediated via the suppression of ROS production [62–64]. In corroboration, blood samples derived from the subjects with BoNT injection have been characterized by a reduced level of ROS [55]. Besides, ample reports experimentally validated the cytoprotective role of BoNT as it counterbalances the increased levels of oxidative stress and free radical production with the elevation of antioxidant enzymes. In a study by Uchiyama et al., BoNT has been reported to reduce ROS in vascular endothelial cells in vitro in cutaneous ischemia–reperfusion (I/R) injuries [22], BoNT type B injection has also been shown to reduce oxidative and endoplasmic reticulum stress induced by I/R injury in a mouse model [21]. Yu et al. demonstrated that the level of malondialdehyde (MDA), a marker of oxidative stress which indicates the rate of lipid peroxidation, was considerably reduced in spinal cord injury-induced mice model treated with BoNT along with minocycline, indicating reduced oxidative stress [65]. Further, many studies have suggested the use of therapeutic BoNT-A in urinary bladder dysfunctions resulting from prostatic hyperplasia due to its positive effect in controlling oxidative stress [66, 67]. Very recently, Yesudhas et al. reported that BoNT improves the enzymatic activities of key antioxidants and provides neuroprotection in the brain of ageing experimental animals [11, 23]. Therefore, BoNT might counteract the ageing-associated excessive release of ACh and regulate the production of testosterone and antioxidant enzymes, thereby facilitating spermatogenesis. Moreover, BoNT treatment could potentially elicit cytotropic signalling events or neutralize the apoptotic signalling cascades in the testis, leading to the increased survival of cellular components of the seminiferous tubules. Taken together, a mild amount of BoNT might be a potential therapeutic aid to treat male infertility.

## Conclusions

Reduced sperm count responsible for male infertility has been a rising medical concern worldwide. The risk for male infertility includes ageing, familial, sporadic and abnormal lifestyle, environmental factors, testicular damage and atrophy, infection, exposure to radiation, chemicals, pesticides, hazardous materials, occupational hazards, alcohol consumption, drug addiction, and disorders with metabolic, vascular, neurological, psychiatric and malignancy complications. Though hormonal replacement, aromatase inhibitors, dopamine antagonists and some surgical procedures have been practiced to rectify male infertility, the efficacy of these treatments have been the subject of debate and often they appear to pose adverse effects than clinical rectifications. Of late, cholinergic toxicity has been proposed as an underlying cause of erectile dysfunction, testicular atrophy and infertility. Recently, blockade of ACh release by BoNT has been used to treat various testicular defects like bilateral cremasteric muscle spasms, retractile testis, cryptorchidism and erectile dysfunction. The present study demonstrates that a mild intramuscular injection of BoNT provides defence against ageing-mediated spermatogenic decline and improves the total sperm count and motility in correlation with increased activities of key antioxidants in the testis of experiment mice. Therefore, BoNT might be considered as a potential drug to treat male infertility. However, possibilities for the unknown adverse effects associated with BoNT might not be completely excluded.

## Methods

### Injection of BoNT in experimental animals

For this study, 7–8-months-old (N = 12) male BALB/c mice were randomly divided into two groups, namely the control group (N = 6) and BoNT treated group (N = 6). A therapeutic form of BoNT (Allergan, Dublin, Ireland) was dissolved in sterile saline. Experimental mice in the test group have received a single intramuscular injection of BoNT at 1U per kilogram (Kg) bodyweight (BW) in the vastus lateralis muscle of the thigh as previously described [11, 23]. An equal volume of sterile saline was injected to each mouse in the control group. Four weeks later, each mouse was sacrificed, the left testis was dissected out and processed for histological examination of spermatogenesis, while the right testis and cauda epididymis were processed for biochemical analysis of antioxidant levels, and sperm analysis respectively. All the experiments were performed in accordance with the approval of the Institutional Animal Ethical Committee (IAEC) under the regulation of the Committee for the Purpose of Control and Supervision of Experimental Animals (CPCSEA), India at Bharathidasan University (Reference No: BDU/IAEC/P272018, Date: 07.08.2018).

### Sperm count and motility

To assess the sperm parameters, the cauda epididymis was removed from each animal and minced in 1% bovine serum albumin (BSA) prepared with 1X phosphate-buffered saline (PBS) at 37 °C. The resulting epididymal fluid was diluted in the same solution (1:4) and placed in the Neubauer chamber. The number of sperms and their degree of motility were estimated using an inverted phase-contrast microscope (DMi1, Leica Microsystems, Germany). The total sperm count was calculated using the formula, Sperm count/ml = Mean sperm count x dilution factor × 10^4^. The motility of sperms was graded as progressive and non-progressive motility and total percentage motility was estimated as previously described [68]. Next, smears of the epididymal fluid were prepared on the microscopic slides (Borosil, India) and stained using a solution containing haematoxylin (H) and eosin (E) (Nice Chemicals, India). The H and E-stained sperms on the slides were fixed and mounted using Dibutylphthalate Polystyrene Xylene (DPX) (Merck, Germany) and dried overnight at room temperature. The morphology and structure of the sperms were evaluated under the light microscope equipped with a camera and the software programme Leica Application Suite (DM750, Leica Microsystems, Germany).

### Histological evaluation of seminiferous tubules and spermatogenesis

The testes were fixed with 10% neutral buffered formalin (NBF), dehydrated using varying concentrations of ethanol (70%, 80%, 90%, 100%) followed by clearing using xylene, after which the tissues were infiltrated and embedded in the paraffin wax. The wax embedded tissues were cut into cross-sections of 10 μm thickness using a rotary microtome (Weswox, India). The testicular sections were placed in silane coated microscopic glass slides and stained with H and E (Nice Chemicals, India). The histological sections were examined and photodocumented using a light microscope. The total number of the cross-section of seminiferous tubules were counted in 10 non-serial cross-sections of the testis in each animal from the control and treatment group. The diameters of the cross-section of seminiferous tubules and lumens were measured as previously described [69]. Further, the total number of primary spermatocytes, secondary spermatocytes, round spermatids, and elongated spermatids were systematically quantified in seminiferous tubules per animal. The data on the estimated number of cells are represented per seminiferous tubule [70].

### Estimation of enzymatic activities of testicular antioxidants

The testicular tissue samples were homogenized with radioimmunoprecipitation assay (RIPA) buffer (Thermo scientific) and centrifuged at 12,000 rpm for 20 min at 4 °C. The supernatant was collected and protein estimation was done by the method described by Lowry et al. [71]. The total protein isolates were subjected to the measurement of key antioxidant enzymes as previously described [11]. In order to measure the activity of SOD, the tissue homogenates were mixed with ice-cold ethanol and chloroform and centrifuged for 15 min at 12,000 rpm and the supernatants were collected. Further, 0.1 M tris buffer (pH 8.2) was added to the supernatant, and the reaction was initiated with the addition of 2.64 mM pyrogallol. As one unit SOD activity represents the amount of protein required for 50% inhibition of pyrogallol autoxidation per minute, the absorbance was measured at 440 nm in a microplate reader (Bio-Rad iMark™). To determine the enzymatic strength of catalase, 0.01 M phosphate buffer solution (pH-7.0) and 0.2 M H_2_O_2_ were added to the testicular protein homogenates. After thorough mixing, 5% potassium dichromate acetic acid reagent was added and the samples were kept in a boiling water bath for 10 min. As the blue colour mixture turn into a green-coloured product of chromate acetate, the absorbance was measured at 570 nm. To measure the activities of GSH, the protein samples were precipitated with 25% trichloroacetic acid (TCA) and spun down at 3,000 rpm for 10 min at 4 °C. The supernatant was mixed with 60 µM 5,5′-dithiobis-(2-nitrobenzoic acid) (DTNB) and 50 mM potassium phosphate buffer (pH-7.4). The absorbance of the resulting yellow-coloured reaction mixture was measured at 412 nm. To assay the activity of GPx, 100 µl of protein samples of testes were mixed with a neutral solution containing 0.32 M phosphate, 4 mM GSH, 10 mM sodium azide, 2.5 mM H_2_O_2_ and 0.8 mM ethylenediaminetetraacetic acid (EDTA). Then the tubes were incubated for 5 min at 37 °C and centrifuged at 3,500 rpm for 15 min. To the supernatant, 0.32 M phosphate solution and 60 µM DTNB reagent were added. GSH solutions corresponding to a concentration ranging between 4 and 20 µg/ml were also prepared and served as known standards. The intensity of yellow colour developed was measured in a microplate reader at 412 nm. Values were expressed as mg of GSH consumed per mg of protein [11].

### Statistical analysis

The values are represented as mean ± standard deviation. Student *t* test was applied to measure the statistical significance using Graph Pad Prism. The significance level was assumed at *P* < 0.05, unless otherwise indicated.

## Abbreviations

ACh: Acetylcholine
BoNT: Botulinum toxin
BW: Body weight
BSA: Bovine serum albumin
CPCSEA: Committee for the Purpose of Control and Supervision of Experimental Animals
DPX: Dibutylphthalate Polystyrene Xylene
DTNB: 5,5′-Dithiobis-(2-nitrobenzoic acid)
EDTA: Ethylenediaminetetraacetic acid
GPx: Glutathione peroxidase
GSH: Reduced glutathione
H and E: Haematoxylin and eosin
HPG axis: Hypothalamic–pituitary–gonadal axis
IAEC: Institutional Animal Ethical Committee
I/R: Ischemia–reperfusion
MDA: Malondialdehyde
NBF: Neutral buffered formalin
PBS: Phosphate-buffered saline
RIPA: Radioimmunoprecipitation assay buffer
ROS: Reactive oxygen species
SOD: Superoxide dismutase
TCA: Trichloroacetic acid

## Declarations

### Availability of data and materials

The datasets generated during and/or analysed during the current study are available from the corresponding author on reasonable request.
